# Implementing Active Assisted Living Technology in the Long-term Care of People Living With Dementia to Address Loneliness: European Survey

**DOI:** 10.2196/45231

**Published:** 2023-06-14

**Authors:** Kübra Beliz Budak, Franziska Laporte Uribe, Franka Meiland, Simone Anna Felding, Sonja Teupen, Johannes Michael Bergmann, Rene Mueller-Widmer, Martina Roes

**Affiliations:** 1 Deutsches Zentrum für Neurodegenerative Erkrankungen e.V. site Witten Witten Germany; 2 Department of Nursing Science Faculty of Health University of Witten/Herdecke Witten Germany; 3 Department of Medicine for Older People Amsterdam Public Health Research Institute Vrije Universiteit Medical Center Amsterdam Netherlands

**Keywords:** loneliness, social isolation, active assisted living technology, long-term care, dementia, Alzheimer, implementation, CFIR

## Abstract

**Background:**

In the lives of people with dementia, loneliness is an important issue with psychological and physical consequences. Active assisted living (AAL) technology has been gaining visibility in the care of persons living with dementia, including addressing loneliness. However, to the best of our knowledge, there is a lack of evidence concerning the factors influencing the implementation of AAL technology within the context of dementia, loneliness, and long-term care (LTC).

**Objective:**

We aimed to identify the familiarity with AAL technology that is promising for addressing loneliness in persons living with dementia in LTC in Europe and the factors influencing AAL technology implementation.

**Methods:**

A web-based survey was developed based on findings from our previous literature review. The Consolidated Framework for Implementation Research guided the development and analysis of the survey. Participants included 24 representatives of Alzheimer Europe member associations from 15 European countries. The data were analyzed using basic statistical methods (descriptive statistics).

**Results:**

The baby seal robot Paro was reported to be the most familiar AAL technology by 19 of 24 participants addressing loneliness in people with dementia living in LTC. Participants from Norway (n=2) reported familiarity with 14 AAL technologies, and participants from Serbia (n=1) reported zero familiarity. It seems that countries that invest less in LTC facilities are familiar with fewer AAL technologies. At the same time, these countries report a more positive attitude toward AAL technology, express a higher need for it, and see more advantages than disadvantages than those countries that invest more in LTC. However, a country's investment in LTC facilities does not seem to be linked to other implementation aspects such as costs, planning, and the impact of infrastructure.

**Conclusions:**

Implementation of AAL technology to address loneliness in dementia seems to be linked to familiarity with the technology in a country as well as national investment in LTC facilities. This survey confirms the literature on higher investment countries’ critical stance in regard to AAL technology implementation to address loneliness in persons living with dementia living in LTC. Further research is needed to clarify the potential reasons why familiarity with more AAL technology does not seem to be directly linked with acceptance, positive attitude, or satisfaction with AAL technology addressing loneliness in persons living with dementia.

## Introduction

Dementia is a growing concern worldwide. The World Health Organization estimates that 10 million people are diagnosed with dementia each year [1]. This growing group of people has specific needs and issues, and one of them is loneliness. Loneliness is defined “as a subjective feeling state of being alone, separated or apart from others and is an imbalance between desired social contacts and actual social contacts” [2,3]. Loneliness among older adults is found to be a factor that may add to the progression of symptoms of dementia and mild cognitive impairment [4]. The predictive power of loneliness on the progression of dementia and MCI is comparable to that of some biological measures, such as higher cortical amyloid burden [5], genetics, diabetes, and vascular diseases [6].

For persons living with dementia, needing to transfer from home to a long-term care (LTC) facility due to disease progression [7] or no longer being able to live safely at home without additional support beyond the care provided by informal caregivers [8] exacerbates the likelihood of loneliness, social isolation, and depression [9]. Loneliness among LTC residents is often addressed by a number of psychosocial interventions, for example, telephone befriending or horticultural therapy [10]. However, the experience of loneliness is largely subjective. As there is no one-size-fits-all approach to addressing loneliness, the need to tailor interventions to suit the needs of individuals is growing [10]. Therefore, adopting technology into the delivery of psychosocial interventions could be seen as an opportunity to address loneliness in the growing group of persons living with dementia. However, it can be a challenge to implement these technologies in LTC settings [11].

Implementation involves a set of planned, intentional activities that aim to put into practice evidence-informed policies and practices in real-world services. The goal of effective implementation is to benefit end users of services—children, youth, adults, families, and communities [12]. Researchers have explored ways to implement technology to aid in the care of persons living with dementia [11]. However, the process of using technology to deliver psychosocial interventions has not often been investigated [13].

In the past 2 decades, there has been an increase in research concerning technology in dementia care [14]. These technological advancements targeted to support persons living with dementia are typically called assistive technology [14]. Assistive technology contains a wide range of technological applications with a potential application to dementia care. These include self-contained devices (eg, tablets, wearables, and personal care robots) and software applications (eg, mobile or web-based apps) [15].

Assistive technology specifically for persons with dementia has been defined as “any item, piece of equipment, product or system driven by electronics, whether acquired commercially, off-the-shelf, modified or customized, that is used to help persons with dementia in dealing with the consequences of dementia” [16]. Assistive technology based on this definition is called active assisted living (AAL) technology [15]. Examples of AAL technology include specialized tablets, wearables, social robots, and integrated smart home systems [15]. AAL technology implementation in real-life practice can still be seen as a challenge [11,16-18], with only a few examples noting some promising insights into the positive impact on loneliness in persons living with dementia [16,18].

A recent review on the implementation factors of social robots in LTC reported a range of barriers, such as complexity, physical accessibility, and cost [17]. The high acquisition and maintenance costs of social robots are one of the primary barriers reported by multiple stakeholders [17]. For example, the average price of a popular robot called Paro is approximately €7000 (a currency exchange rate of 1€=US $1.08) [19], and with funding for LTC facilities ranging from one country to another, those costs can be a barrier [20]. The authors of the review also reported that the beliefs and attitudes of stakeholders present an important barrier to the implementation of AAL technology [17]. The authors noted that there is a scarcity of studies that have explored the perceptions of key stakeholders in LTC, such as care professionals, family, and persons living with dementia, even though it is known that these stakeholders play important roles in the implementation process of technology in LTC [17]. Therefore, an understanding of these stakeholders’ perspectives and experiences is needed to bridge the knowledge gap between research and clinical practice.

To the best of our knowledge, there is a lack of evidence on the range of factors affecting AAL technology implementation in the context of loneliness and dementia. Therefore, this study aimed to identify the influencing factors that hinder or facilitate the implementation of AAL technology for residents with dementia in LTC across Europe and the potential impact of such technology on feelings of loneliness. We focus on countries in Europe for the need to bridge the gaps between dementia care across Europe where different sectors have resulted in a patchwork of approaches to technology without a coherent model while competing with rapid advances in the world [21,22]. Specifically, we were interested in answering the following research questions: (1) How do the European Alzheimer association’s view factors that affect the implementation of AAL technology in LTC facilities to address loneliness in persons living with dementia in their respective regions? (2) What is the perspective of dementia associations on the AAL devices that have been implemented in LTC facilities in their region to address loneliness in persons living with dementia? (3) Are there any other factors regarding AAL technology implementation in LTC that might have potential influence?

## Methods

### Overview

The paper reports on a web-based survey consisting of a quantitative questionnaire combined with open-ended questions. Participants were asked fifteen 5-point Likert-scale questions and open-ended questions based on Damschroder “Consolidated Framework for Implementation Research” (CFIR) [21]. The web-based survey tool (LimeSurvey) was used.

### Participants

The participants included stakeholders who were experts from European national and regional Alzheimer associations and thus were knowledgeable about the use of AAL technology in their respective country or region. We reached out to Alzheimer associations in 47 European countries, but we were able to contact only 34 Alzheimer associations. All were members of Alzheimer Europe, which is the most comprehensive collaboration of Alzheimer associations in Europe.

We aimed to determine which implementation barriers are experienced in each region and which are perceived to be more relevant to loneliness. Thus, we addressed national and regional Alzheimer associations and inquired whether they were familiar with the general trend of the overall beliefs and attitudes of stakeholders toward the use of AAL technology in LTC facilities. We believe that surveying European Alzheimer associations provided a more comprehensive outlook of the region, whereas asking individual LTC facilities may have resulted in points that could not be generalized.

For this web-based survey, representatives from 34 National Alzheimer associations of Alzheimer Europe were informed about the study and invited to participate in the survey via a personalized email. With this initial email contact, Alzheimer association representatives were also asked to forward the survey information to their regional Alzheimer association contacts. The researcher (KBB) identified 34 Alzheimer associations’ contacts through publicly available information on their respective websites.

The inclusion criterion for participants was that they spoke sufficient English to understand the study information and complete the web-based survey. Alzheimer associations were given the opportunity to register and provide written informed consent via email. The participants were asked to respond to the survey within 21 days, with email reminders sent every 5 days, and the survey was closed after 28 days. Another reminder was sent out within the extended deadline to solicit additional completed surveys.

Following the first descriptive analyses, we decided to include national LTC expenditure, that is, national funds invested in LTC facilities, as a factor for implementation. We used the data from the Organisation for Economic Co-operation and Development (OECD) to see the national expenditure on LTC facilities measured with current prices in Euro (€) [20]. Consequently, 2 groups of participating countries formed according to their national expenditure on LTC facilities, namely, higher and lower expenditure groups.

The respondents’ countries are as follows: Portugal, Germany, Belgium, France, Netherlands, Norway, Bulgaria, Czech Republic, Finland, Greece, Luxembourg, Malta, Serbia, Slovenia, and Switzerland. Next, we grouped the countries by annual national expenditure on LTC facilities, yielding lower- and higher expenditure groups. National expenditure is the amount of capital invested in LTC facilities by government/compulsory and private/out-of-pocket budgets [23]. Lower and higher expenditure groups (per capita) are defined by the latest OECD values available for all participating countries, which were from 2019 (Multimedia Appendix 1) [24].

### Methodological Framework

We used the CFIR [25] to identify barriers to and facilitators of AAL technology implementation. We chose the CFIR because it provides a useful structure for identifying potential factors influencing implementation at multiple levels [25]. The CFIR includes 39 constructs (ie, determinants) organized into 5 domains: innovation characteristics (eg, complexity and strength of the evidence), outer setting (eg, external policy and incentives), inner setting (eg, organizational culture and the extent to which leaders are engaged), characteristics of individuals involved (eg, self-efficacy using AAL technology in a sustainable way), and process (eg, planning and engaging key stakeholders) [25,26]. All constructs interact to affect the process and effectiveness of implementation [25,27]. Therefore, using this framework enables the identified barriers and facilitators to be presented in a structured and systematic manner. It also allows findings to be easily compared to those of other implementation studies to identify research gaps.

### Design of the Web-Based Survey

Damschroder et al [25] recommended that researchers try to identify CFIR constructs early on, assess them based on their relevance to the study, and then determine at what level each construct should be measured. In our scoping review [3], we identified 10 of the 39 CFIR constructs as relevant in implementing AAL technology to address loneliness in persons living with dementia in LTC and therefore relevant for our web-based survey:

1. Intervention characteristics
  Relative advantage: Q1 and Q3
  Cost: Q5
2. Outer setting
  Patient needs and resources: Q4 and Q6
  External policies and incentives: Q13
3. Inner setting
  Structural characteristics: Q7 and Q8
  Culture: Q9
  Implementation climate
    Tension for change: Q2
    Compatibility: Q10 and Q11
    Relative priority: Q12
4. Process
  Planning: Q14
  Engaging
    Key stakeholders: Q15

Our team reviewed and revised the survey, and the final version had 15 questions. Questions were adapted from the CFIR interview guide from the relevant 10 domains identified by the scoping review. Then, we reviewed the survey and answer choices with an English language expert to ensure suitability for nonacademic staff in Alzheimer associations. Furthermore, we asked 3 Alzheimer associations to pretest and validate the survey for suitability. Two of them responded and gave detailed feedback. We revised the survey accordingly. The survey was then designed in a web-based survey tool (LimeSurvey) [28] and tested for any technical issues.

The web-based survey questionnaire, the recruitment plan, and the deployment plan were extensively discussed with Alzheimer Europe, with whom the researcher (KBB) worked closely using a participatory approach within the project DISTINCT (Dementia: Intersectorial Strategy for Training and Innovation Network for Current Technology), where this study was funded [21]. Alzheimer Europe was involved because they are the most comprehensive union of Alzheimer associations in Europe, and they are one of the collaborative partners of the DISTINCT consortium, which is an EU-funded Marie Skłodowska-Curie research and training project.

### Ethics Approval

This study received ethical approval from the University of Witten/Herdecke with approval number SR-205/2021. This survey was conducted with ethical principles in mind. In accordance with recommendations for good internet-based research by Gupta [29], the participants were shown an information form and were asked to provide consent before they were able to see the questionnaire. The information form provided complete details of the study, including contact information, study aims, data collection procedure, potential benefits and harms, and steps taken to maintain the anonymity and confidentiality of the participants. These steps enabled the participants to reach out to the investigators and clarify whether they had any questions or concerns. Cookies were used to prevent accessing the survey twice. No personal information about the participants was collected. Survey data were saved in a secure server upon completion and were accessible only to the first author. The participants were informed that they could request to opt-out at any time and could request to delete their records. More detailed information about this process can be obtained from the CHERRIES (Checklist for Reporting Results of Internet E-Surveys) checklist in Multimedia Appendix 2.

### Analysis

Descriptive statistics were generated using SPSS (IBM Corp) and Excel (Microsoft) [30,31]. The visualization of the data by the balloon plots was performed with the statistical software R (R Foundation) [32], and graphical representations of the data were created with the package ggplot2 [33]. Due to the low response rate (50%), the available data were analyzed using basic statistical methods, and descriptive statistics were calculated. CFIR was used to guide the analysis process [25].

## Results

### Participants

This survey yielded 24 full responses across 15 European countries from the 34 national and regional Alzheimer associations across 30 European countries (see Table 1) that were contacted, for a response rate of 50%. Thirty national and regional associations were contacted and 15 responded (15/30×100). Organizations in Austria, Croatia, Cyprus, United Kingdom, Denmark, Estonia, Hungary, Ireland, Iceland, Italy, Jersey, Poland, Romania, Slovakia, Spain, Sweden, and Scotland were contacted but did not respond to the survey. For this purpose, we considered both national and regional Alzheimer associations. Therefore, 4 regional responses were added to the national responses.

Two of the participants reported their age group as 18-30 years, 12 participants were between the ages of 31 and 50 years, and 10 were older than 50 years. Seventeen participants were female, 6 were male, and 1 participant was nonbinary. Eleven participants were from national Alzheimer associations, whereas 13 participants were from regional Alzheimer associations. The highest number of responses from 1 country came from Portugal (n=4), followed by Germany (n=3) and then Belgium, France, the Netherlands, and Norway (n=2). The remaining responses came individually (n=1) from the following countries: Bulgaria, Czech Republic, Finland, Greece, Luxembourg, Malta, Serbia, Slovenia, and Switzerland.

**Table 1:** Participating Alzheimer associations.

			How many answered		National expenditure in LTC^a^ per capita (€)^b^
**Answered from a national Alzheimer association**					
	Bulgaria	1		1.3	
	Czech Republic	1		188	
	France	1		417.7	
	Germany	1		424.6	
	Luxemburg	1		340	
	Malta	1		523.3	
	Netherlands	1		1047	
	Norway	2		705.1	
	Portugal	1		28.7	
	Slovenia	1		147.2	
**Answered from a regional Alzheimer association**					
	Belgium	2		474.4	
	Finland	1		363.5	
	France	2		417.7	
	Germany	2		424.6	
	Greece	1		20.6	
	Netherlands	1		1047	
	Portugal	3		28.7	
	Serbia	1		N/A^c^	
	Switzerland	1		816.6	

^a^LTC: long-term care.

^b^A currency exchange rate of 1€=US $1.08.

^c^N/A: not applicable.

### Perceived Familiarity With AAL Technology Across Europe

#### Overview

Perceived familiarity is considered a factor affecting the implementation of AAL technology to address loneliness in persons living with dementia. The following sections on perceived familiarity are structured based on types of AAL technology (see Multimedia Appendix 3 for an overview).

#### Familiarity of AAL Technology and Social Robots

The participants were asked whether they were familiar with the following type of AAL technology with regard to addressing loneliness in persons living with dementia: pet robots, for example, “Paro”; humanoid robots, for example, “Pepper”; multimedia computer systems, for example, “Xbox”; and telepresence robots, for example, “Giraff.” The most familiar pet robot was the baby seal robot “Paro” (n=19), followed only by the “Joy for All” cat (n=6), while 6 participants were not familiar with any pet robots (n=6). Ten of the 15 countries reported having Paro. Humanoid robots were less popular, and the respondents were most familiar with Pepper, as reported by 7 countries. Papero was reported only in Czech Republic, and Cuddler was reported only in Finland. More countries were unfamiliar with humanoid robots than pet robots; 9 of the 15 countries were not familiar with them.

#### Open-Ended Questions

The participants were asked to manually report any other AAL technology in case the devices that they were familiar with were not on the list. These data are presented as a list of technologies the participants were familiar with (Table 2). One technology that was identified was “Tovertafel.” A gesture-controlled multimedia table was reported by 3 participants, and “KOMP,” a simple tablet computer for video-calling, was reported by 2 participants. The remaining answers were “smart assistants” such as “Siri or Alexa”; “BeleefTV,” a touchscreen on wheels with sensory games and reminiscence; the “Cogweb,” computer system that provides cognitive exercises; “Smartmacadam,” an app for daily planning; “Music doll,” a therapy doll with a built-in music player; “Easy music player”; and the “Motitech” stationary bike with video. We also asked the participants how they became familiar with these technologies, for example, having direct knowledge of their implementation or having heard about them from other regions or countries. Six participants reported that the technology was actively implemented in their regions, and 5 reported demonstrations by the manufacturers, while 1 participant did not report their source of knowledge.

**Table 2:** Pet and humanoid robots in Europe.

Country	Paro	Aibo	JustoCat	JoyforAll Cat	JoyforAll Dog	Papero^a^	Pepper^a^	Cuddler
Belgium	✓		✓	✓	✓		✓	
Bulgaria								
Czech Republic						✓		
Finland							✓	✓
France	✓			✓				
Germany	✓		✓	✓	✓		✓	
Luxemburg	✓			✓				
Malta	✓							
Netherlands	✓						✓	
Norway	✓	✓	✓	✓			✓	
Portugal	✓							
Slovenia	✓						✓	
Switzerland	✓	✓					✓	
Serbia								
Greece								

^a^Humanoid robots.

#### Familiarity With Multimedia Computer Systems

Fourteen participants were not familiar with any of the multimedia computer systems, whereas 9 participants were familiar with the Digital Lifestorybook. None of the countries were familiar with CIRCA, VENSTER, or ChitChatters. Digital Lifestorybook, on the other hand, was familiar to the respondents in 9 out of 15 countries. Nevertheless, 11 countries reported being unfamiliar with multimedia computer systems. On the other hand, more countries (n=12) were familiar with Nintendo Wii and Xbox than any other options. Although 10 countries were familiar with PlayStation, only 6 countries were unfamiliar with any of the systems (Table 3).

**Table 3:** Multimedia computer systems by country.

Country	Digital Lifestorybook	Nintendo Wii	Xbox	PlayStation
Belgium	✓	✓	✓	✓
Bulgaria				
Czech Republic	✓	✓	✓	✓
Finland	✓		✓	
France	✓	✓		
Germany	✓	✓	✓	✓
Luxemburg	✓	✓	✓	✓
Malta		✓	✓	✓
Netherlands		✓	✓	✓
Norway	✓	✓	✓	✓
Portugal	✓	✓	✓	✓
Slovenia		✓	✓	
Switzerland			✓	✓
Serbia	✓			
Greece		✓	✓	✓

#### Familiarity With Telepresence Robots and Other Technology

Most participants (n=20) reported no familiarity with telepresence robots, with some (n=3) participants familiar with Giraff. The respondents from 13 of 15 countries reported being unfamiliar with telepresence robots. CompanionAble was unknown by all respondents, and the Guide was reported by only 1 participant. Other technologies were reported to be actively implemented only in certain countries. “Cogweb” was reported to be actively implemented in Portugal, “Tovertafel” was reportedly implemented in some care homes in Germany, and “Motitech,” “KOMP,” “The music doll,” and “Easy music player” were reported to be popular in LTC facilities in Norway. Smartmacadam was reported only in France.

### Factors Influencing the Implementation of AAL Technology and Social Robots Across Europe

#### Overview

**Figure 1:**
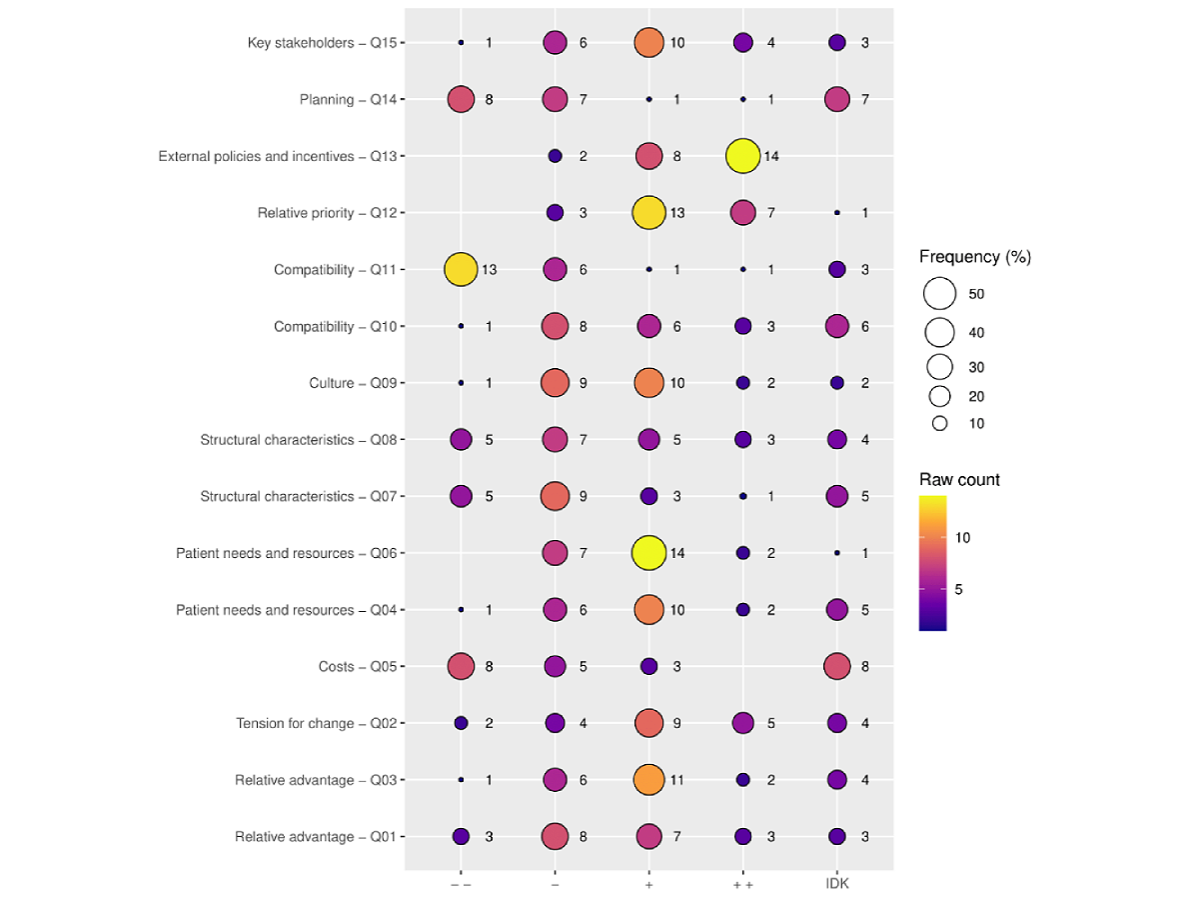
Survey results by Consolidated Framework for Implementation Research (CFIR) domains. Statistical frequencies displayed by survey questions and CFIR domains. (− −): highly negative; (−): slightly negative; (+): slightly positive; (++): highly positive; IDK: I don’t know.

The participants were asked 15 multiple-choice questions on the factors affecting the implementation of AAL technology in LTC facilities regarding loneliness in persons living with dementia. All questions were fully answered by all participants (n=24). In the following section, the results are presented according to the CFIR implementation domains (see Figure 1).

#### Innovation Characteristics—Relative Advantage

**Figure 2:**
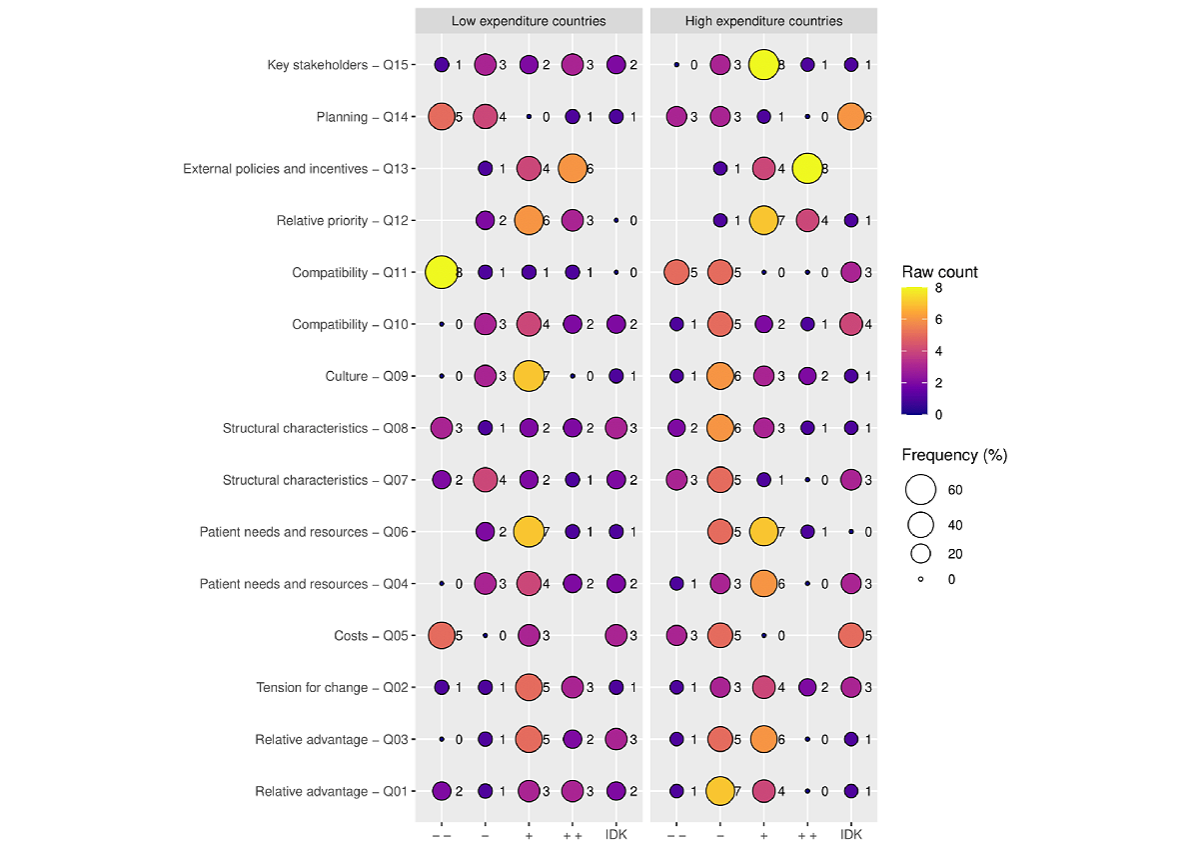
Low- and high-expenditure countries’ differences by Consolidated Framework for Implementation Research domains. Frequencies displayed by 2 groups divided by national expenditure on long-term care. Notable differences between low- and high-expenditure countries in Q1: relative advantage; Q2: tension for change; Q3: relative advantage; Q5: costs; Q8: structural characteristics; Q9: culture; Q15: key stakeholders. (− −): highly negative; (−): slightly negative; (+): slightly positive; (++): highly positive; IDK: I don’t know; Q: survey question.

The participants pointed out that the team atmosphere in an LTC facility influences how care professionals perceive AAL technology and its impact on loneliness (slightly positive=7, slightly negative=8). The team atmosphere concerning the use of AAL technology was reported to be *slightly more positive* in countries in the lower expenditure group (see Figure 2). Countries in the lower expenditure group reported more advantages of AAL technology in LTC facilities than countries in the higher expenditure group.

#### Innovation Characteristics—Cost

Eight participants reported that AAL technology aimed at loneliness comes with *high additional costs*, and another 8 reported that *they are not aware of the costs*. No participants reported that they had *no additional costs*. The higher expenditure group reported slightly more *noticeable additional costs*, while the lower expenditure group noted slightly more *high additional costs*.

#### Outer Setting—Patient Needs and Resources

Ten participants revealed a *somewhat positive attitude* among persons living with dementia about AAL technology addressing loneliness, whereas 6 noted a *somewhat negative* attitude among persons living with dementia. Fourteen participants reported that AAL technology meets *some needs and preferences of* persons living with dementia, and this response was shared by most participants, making it one of the most agreed-upon subdomains. No participants reported that AAL technology *meets no needs at all*. The respondents in the lower expenditure group reported a slightly more *positive attitude* toward persons living with dementia than those in the higher expenditure group. Again, those in the lower expenditure group reported a slightly more *positive* response regarding the ability of AAL technology to meet more needs than those in the higher expenditure group.

#### Outer Setting—External Policies and Incentives

Fourteen participants responded that external financial support would *definitely increase* AAL technology use to address loneliness in persons living with dementia. This response was given by most participants, making it one of the most agreed-upon subdomains. No participants reported that financial support would not increase AAL technology use or that they did not know. Countries in the higher expenditure group reported *slightly higher* chances of an increase in AAL technology use than those in the lower expenditure group.

#### Inner Setting—Structural Characteristics

Nine participants revealed that the infrastructure of the LTC facilities corresponds *slightly negatively* to AAL technology implementation aimed at addressing loneliness, and 5 noted that it corresponds *highly negatively*. Seven participants answered that there were *some building plan changes* necessary to implement AAL technology, and 5 noted that *many building plans* were necessary. The participants from countries in the lower expenditure group reported that the infrastructure of their regions had a slightly more *positive* impact on implementation than the participants from countries in the higher expenditure group. Additionally, the participants indicated whether they needed any infrastructure changes to implement AAL technology, and the lower expenditure group reported that *fewer changes were necessary* than the higher expenditure group.

#### Inner Setting—Culture

Ten participants noted that LTC culture corresponds *slightly positively* with the implementation of AAL technology addressing loneliness, and 9 reported a *slightly negative* correspondence. Two added that they were not aware, 1 reported that it corresponds *highly negatively*, and 2 reported *highly positively*. The lower expenditure group reported that culture corresponds slightly more positively with implementation than the higher expenditure group.

#### Inner Setting Implementation Climate—Tension for Change

The participants reported how stakeholders, for example, care professionals, persons living with dementia, and informal caregivers, consider the need for AAL technology for persons living with dementia in their region to help decrease loneliness. Nine reported a *considerable need*, 4 reported *only a little need*, and another 5 reported that there was a *strong need*. Countries in the lower expenditure group reported that more AAL technology is needed than those in the higher expenditure group. Countries in the lower expenditure group reported that AAL technology was *needed slightly more* than countries in the higher expenditure group.

#### Inner Setting Compatibility

The participants reported how well AAL technology fits with the values and norms of stakeholders, for example, care professionals and persons living with dementia in LTC, to address loneliness in persons living with dementia. Eight participants noted that AAL technology *does not truly fit* the stakeholders’ values and norms, 6 revealed that they were not aware, and another 6 noted that AAL technology *somewhat fits their values and norms*. Thirteen participants reported that AAL technology *did not replace* any nontechnological interventions for loneliness, and 6 noted that it *did not really replace* any interventions. Countries in the lower expenditure group reported *higher fit* than countries in the higher expenditure group. When asked whether AAL technology replaced any existing programs for loneliness, both groups reported *no replacement*.

#### Inner Setting—Relative Priority

When asked about the importance of AAL technology aimed at addressing loneliness compared to other priorities, such as fall prevention, 13 of the participants responded that it was *somewhat important* in comparison to other priorities in the LTC facility. Seven reported that it was *very important*, 3 declared that it was *not really important*, and 1 did not know. No participants reported that AAL technology is *not at all important*. There were no differences between the lower and higher expenditure groups.

#### Process—Planning

Eight of the participants noted that LTC facilities in their area had *no plan in place at all* to implement AAL technology to address loneliness in their region; 7 had *hardly any plan in place*, 1 had a *partial plan*, and another had *considerable plans* in place. Seven participants were not aware. Lower expenditure countries reflected that they had fewer plans than higher expenditure countries.

#### Process—Engaging Key Stakeholders

Ten participants reported that Alzheimer associations provided *some encouragement* to the LTC facilities in their regions to implement AAL technology to address loneliness in persons living with dementia, 6 reported that the associations *did* *not really encourage* LTCs in their area, 4 reported that they *encouraged them highly*, 1 reported that they *did not encourage LTCs at all*, and 3 reported no knowledge. The participants were asked whether Alzheimer associations encouraged LTCs in their regions to implement AAL technology, and the higher expenditure group reported *slightly higher* encouragement than the lower expenditure group.

### Additional Factors

According to OECD data [23], national expenditure on LTC facilities appears to be a factor that might mitigate the familiarity of AAL technology in a given country or region. It also appears that Northwestern European countries are familiar with more AAL technology than Southeastern European countries. This can be observed in the expenditure on LTC facilities (see Multimedia Appendix 1).

## Discussion

### Principal Findings

In this survey, we investigated familiarity with AAL technology in Europe and the factors influencing the implementation of AAL technology in LTC. We have found that the seal pet robot Paro was the most familiar AAL technology being used to address loneliness in persons living with dementia across Europe. Pet robots were more familiar than other types of AAL technologies. The least familiar AAL technology was telepresence robots. Survey respondents were on average familiar with 7 AAL technologies, ranging between 14 and zero.

### Comparison to Prior Work

The literature suggests an array of implementation barriers when implementing AAL technology in LTC to address loneliness in persons living with dementia, such as user capabilities, user willingness, and family support [34]. In this paper, we investigated the perceptions of European Alzheimer associations regarding implementation barriers. Those countries in the lower expenditure group appeared more accepting toward AAL technology implementation in LTCs, in accordance with previous research [35]. At the same time, the respondents from these countries reported being familiar with fewer technologies. The participants from countries in the higher expenditure group generally reported less acceptance, more disadvantages, less fit with norms and values, and less interest from persons living with dementia. This group also reported being familiar with a higher number of technologies. It seems that the fewer technologies a country has, the higher the interest in more technology. This may be due to the active experience of the implementation phase, where care professionals experience the implementation barriers first hand. For example, in Spain, a lower expenditure country, it was found that the effective use of AAL technology could allow care professionals to spend more time on social intervention and less on administrative tasks [35]. Despite hardships, care professionals displayed an optimistic point-of-view toward AAL technology [35]. However, in Norway, implementation of a social communication tool (KOMP) changed work routines and created additional responsibilities for care professionals [36]. Even though care professionals tried to come up with creative ways to motivate persons living with dementia, using KOMP was limited by the physical and cognitive abilities of its users [36]. This is a novel finding considering the lack of studies in the literature focusing on lower expenditure countries [35] and a number of studies focusing on higher expenditure countries [34,36,37].

In Germany, a higher expenditure country, lower acceptance of Giraff in persons living with dementia was found to be linked to the lower psychological well-being and lower cognitive abilities of residents, unlike the case of Paro [38,39]. This could be explained by the lower cognitive requirements of using Paro as opposed to using more complicated technologies such as Giraff. Additionally, in Germany, using digital technology for social engagement increased 72.8% (n=349) during the COVID-19 pandemic [34]. However, relatively slow uptake of the technology by residents with dementia due to the absence of adequate support from staff and the lack of staff training were indicated as barriers to implementation alongside costs [34]. This confirms our finding on the critical stance of higher expenditure countries toward AAL technology not meeting many needs of residents and not fitting with existing workflows. In the United Kingdom, a high-expenditure country, technological illiteracy, the low technological confidence of staff, and persons living with dementia being distressed by the robotic voice of the device were reported as barriers to implementing smart speakers in LTC facilities [40]. In Ireland, another high-expenditure country, interviews with care professionals revealed that costs, lack of personnel, and concerns about meeting the needs of persons living with dementia were perceived barriers to the implementation of social robots in LTC facilities [41].

The findings of a review that included studies from the United Kingdom, the Netherlands, Finland, and Sweden suggest that persons living with dementia can potentially benefit from using digital technologies such as videoconferencing to help them maintain and create social networks [37]. However, the usability of the technology, supporting individuals in using the technologies, and training the family caregivers are issues that need further research [37]. Higher expenditure countries seem somewhat more experienced but also more critical toward the implementation of AAL technology. Therefore, this survey confirms the literature findings on the higher expenditure countries’ critical stance on AAL technology implementation due to, but not limited to, concerns about costs, staff resources, the technological illiteracy of staff, and the cognitive abilities of residents.

Based on our findings, we argue that there is no direct link between the funds spent on AAL technology in LTC and the satisfaction gained from it for both residents and staff. The technological literacy of both staff and residents seems to play an important role in implementation. Care practitioners need to match the needs of the residents and staff with the functionality of the AAL technology. Policy makers are likely to benefit from facilitating a dialog with stakeholders involved in co-design [42] and co-research [43] efforts in dementia and in AAL technology, approaches that are becoming increasingly relevant both in practice and in research.

### Study Limitations

First, the staff involved in implementing AAL technology in nursing homes were not included in the sample. This may have affected the results in such a way that we might not be informed about the first-hand experiences of AAL technology implementation. The survey inquired about only participants’ perspectives on factors potentially influencing AAL technology implementation and the status quo of AAL technology implementation in relation to addressing loneliness in persons living with dementia based on their expertise as regional or national dementia organizations. Including LTC facilities in different European countries in the web-based survey could have provided first-hand perspectives on the implementation of AAL technologies, in addition to the broader picture provided by Alzheimer associations. However, the expected effort needed to obtain contact data, translate the survey into different national languages, and obtain a representative sample of LTCs outweighed the expected added value given the available time and human resources.

In addition, the original names of the AAL technologies were used as in the scoping review prior to the survey. However, the authors point out that some of the technology might have been known by other names in the field of LTC. For example, a multimedia technology, ChitChatters, is also known by the Dutch name “de Klessebessers.” This might have impacted the familiarity of the respondents with this particular AAL technology.

Additionally, familiarity with AAL technology was explored in the context of addressing loneliness in persons living with dementia in LTC, whereas the survey respondents might have considered a certain AAL technology as familiar for other reasons, such as personal usage.

Furthermore, the respondents from 5 of the 7 countries in the lower expenditure group reported familiarity with the multimedia computer systems Xbox, PlayStation, and Nintendo Wii. This result must be taken with caution due to the worldwide availability of these devices for various contexts. These devices are known throughout the world for video gaming, and there are no data showing that they are known in LTC settings specifically and not from personal entertainment experience.

Finally, the sample size was small (N=24), which can be seen as a factor that limits the generalizability of the results. It was also somewhat unbalanced, with some countries being more represented than others. The same applies to the representation of national and regional associations within the countries.

### Conclusions

Paro was found to be the most familiar AAL technology, and telepresence robots were the least familiar. Northwestern European countries were familiar with more devices than Eastern and Southern European countries. This finding corresponds with the national LTC expenditures of participating countries [20].

It seems that the expenditure of European countries on LTC facilities might be linked with the number of AAL technologies their citizens are familiar with. The respondents from higher expenditure countries reported that they encourage their associations to implement AAL technology in their areas more than those in the lower expenditure group, despite their critical stance toward AAL technology. Future research is needed to clarify the potential reasons why LTC expenditure is not linked with acceptance, attitudes, or satisfaction with AAL technology in LTC.

European Alzheimer associations generally seem to agree that AAL technology meets only some needs and preferences of persons living with dementia; that AAL technology is somewhat more important than other priorities in LTC facilities, such as fall prevention; and that external financial support would increase AAL technology use to address loneliness in persons living with dementia.

Finally, the attitude of stakeholders seems to have a more positive impact in lower expenditure countries. Therefore, further research is needed to extend and diversify the role of AAL technology in addressing loneliness in persons living with dementia.

## Appendix

Multimedia Appendix 1 Participant countries' long-term care (LTC) expenditure per capita.

Multimedia Appendix 2 CHERRIES (Checklist for Reporting Results of Internet E-Surveys).

Multimedia Appendix 3 Active assisted living technology per country.

## Abbreviations

AAL: active assisted living
CFIR: Consolidated Framework for Implementation Research
CHERRIES: Checklist for Reporting Results of Internet E-Surveys
DISTINCT: Dementia: Intersectorial Strategy for Training and Innovation Network for Current Technology
LTC: long-term care
OECD: Organization for Economic Cooperation and Development
