# Supplementary figures and images for: Implementing Active Assisted Living Technology in the Long-term Care of People Living With Dementia to Address Loneliness: European Survey
Source: JMIR Aging. 2023 Jun 14;6:e45231. doi: 10.2196/45231 (PMC10334712; doi:10.2196/45231)

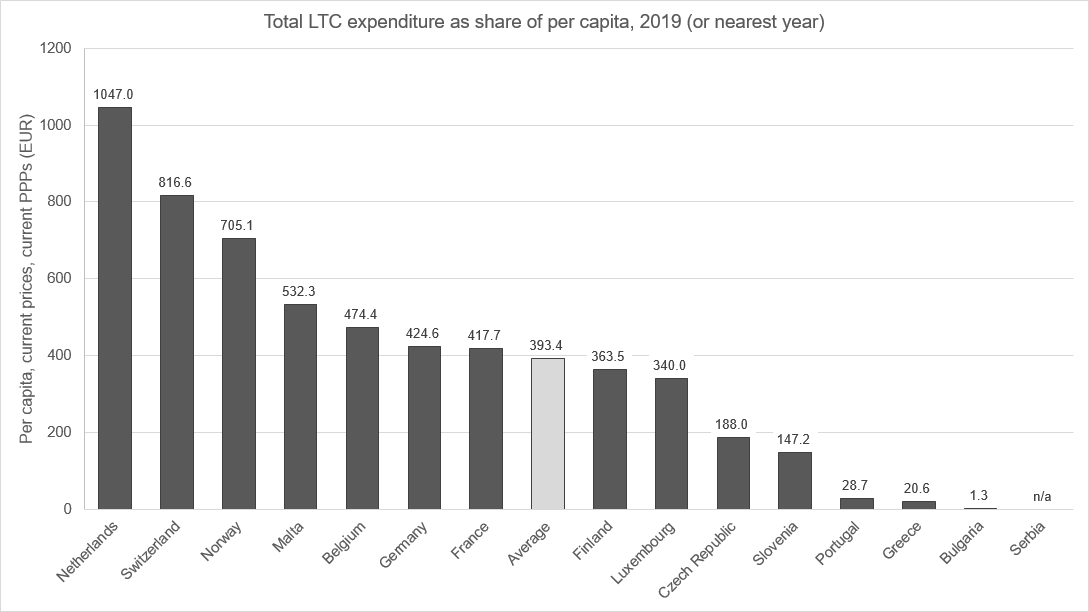

Supplement: Multimedia Appendix 1 [file aging_v6i1e45231_app1.png]
