# Supplementary material for: Implementing Active Assisted Living Technology in the Long-term Care of People Living With Dementia to Address Loneliness: European Survey
Source: JMIR Aging. 2023 Jun 14;6:e45231. doi: 10.2196/45231 (PMC10334712; doi:10.2196/45231)
Supplement: Multimedia Appendix 2 [file aging_v6i1e45231_app2.pdf]

Table 1. Checklist for Reporting Results of Internet E-Surveys (CHERRIES)

	**Checklist for Reporting Results of Internet E-Surveys (CHERRIES)**		
Item Category	***Checklist Item***	***Explanation***	***Author Response***
**Design**			
	Describe survey design	Describe target population, sample frame. Is the sample a convenience sample? (In “open” surveys this is most likely.)	Targeted survey for European Alzheimer Associations.
**IRB (Institutional Review Board) approval and informed consent process**			
	IRB approval	Mention whether the study has been approved by an IRB.	Ethical approval from the University of Witten/Herdecke,approval number: SR-205/2021.
	Informed consent	Describe the informed consent process. Where were the participants told the length of time of the survey, which data were stored and where and for how long, who the investigator was, and the purpose of the study?	The participants were shown the information form and asked to provide consent before they could see the questionnaire. The information form provided complete details of the study, including contact information, study aims, data collection procedure, potential benefits and harms, and steps taken to maintain anonymity and confidentiality of the participants. Participants were informed that they could have requested to opt out any time and that they could have requested to have their records deleted.

	**Checklist for Reporting Results of Internet E-Surveys (CHERRIES)**		
Item Category	***Checklist Item***	***Explanation***	***Author Response***
	Data protection	If any personal information was collected or stored, describe what mechanisms were used to protect unauthorized access.	Anonymous survey, no personal data was stored.
Development and pre-testing			
	Development and testing	State how the survey was developed, including whether the usability and technical functionality of the electronic questionnaire had been tested before fielding the questionnaire.	Please see section “Design of the online survey”
Recruitment process and description of the sample having access to the questionnaire			
	Open survey versus closed survey	An “open survey” is a survey open for each visitor of a site, while a closed survey is only open to a sample which the investigator knows (password-protected survey).	Closed survey; participants were contacted directly through their work emails.
	Contact mode	Indicate whether or not the initial contact with the potential participants was made on the Internet. (Investigators may also send out questionnaires	Initial contact with participants was made via e-mail.

	**Checklist for Reporting Results of Internet E-Surveys (CHERRIES)**		
Item Category	***Checklist Item***	***Explanation***	***Author Response***
		by mail and allow for Web-based data entry.)	
	Advertising the survey	How/where was the survey announced or advertised? Some examples are offline media (newspapers), or online (mailing lists – If yes, which ones?) or banner ads (Where were these banner ads posted and what did they look like?). It is important to know the wording of the announcement as it will heavily influence who chooses to participate. Ideally the survey announcement should be published as an appendix.	Alzheimer Europe informed their national and regional associations in an internal online newsletter.
Survey administration			
	Web/E-mail	State the type of e-survey (eg, one posted on a Web site, or one sent out through e-mail). If it is an e-mail survey, were the responses entered manually into a database, or was there an automatic method for capturing responses?	Online survey using an online survey tool. Please refer to p.7 "Design of the survey" for detailed information.
	Context	Describe the Web site (for mailing list/newsgroup) in which the survey was posted. What is the Web site about, who is visiting it, what are visitors normally looking for? Discuss to	n/a

	Checklist for Reporting Results of Internet E-Surveys (CHERRIES)		
Item Category	***Checklist Item***	***Explanation***	***Author Response***
		what degree the content of the Web site could pre-select the sample or influence the results. For example, a survey about vaccination on a anti-immunization Web site will have different results from a Web survey conducted on a government Web site	
	Mandatory/voluntary	Was it a mandatory survey to be filled in by every visitor who wanted to enter the Web site, or was it a voluntary survey?	Voluntary survey for addressed participants.
	Incentives	Were any incentives offered (eg, monetary, prizes, or non-monetary incentives such as an offer to provide the survey results)?	no
	Time/Date	In what timeframe were the data collected?	13.01.2022-13.02.2022
	Randomization of items or questionnaires	To prevent biases items can be randomized or alternated.	n/a
	Adaptive questioning	Use adaptive questioning (certain items, or only conditionally displayed based on responses to other items) to reduce number and complexity of the questions.	n/a
	Number of Items	What was the number of questionnaire items per page? The number of items is an important	35 items in total, presented in 3 Blocks of 6, 15 and 4 items

	Checklist for Reporting Results of Internet E-Surveys (CHERRIES)		
Item Category	***Checklist Item***	***Explanation***	***Author Response***
		factor for the completion rate.	
	Number of screens (pages)	Over how many pages was the questionnaire distributed? The number of items is an important factor for the completion rate.	5 screens, 3 for presentation of the items
	Completeness check	It is technically possible to do consistency or completeness checks before the questionnaire is submitted. Was this done, and if "yes", how (usually JAVAScript)? An alternative is to check for completeness after the questionnaire has been submitted (and highlight mandatory items). If this has been done, it should be reported. All items should provide a non-response option such as "not applicable" or "rather not say", and selection of one response option should be enforced.	Mandatory questions were highlighted and checked before submit. A non-response option was set in all questions, like "I don't know." Or "I'm not familiar with one of these".
	Review step	State whether respondents were able to review and change their answers (eg, through a Back button or a Review step which displays a summary of the responses and asks the respondents if they are correct).	Respondents were able to go back through a button.
Response rates			

	Checklist for Reporting Results of Internet E-Surveys (CHERRIES)		
Item Category	***Checklist Item***	***Explanation***	***Author Response***
	Unique site visitor	If you provide view rates or participation rates, you need to define how you determined a unique visitor. There are different techniques available, based on IP addresses or cookies or both.	Participant rate based on cookies.
	View rate (Ratio of unique survey visitors/unique site visitors)	Requires counting unique visitors to the first page of the survey, divided by the number of unique site visitors (not page views!). It is not unusual to have view rates of less than 0.1 % if the survey is voluntary.	50/62
	Participation rate (Ratio of unique visitors who agreed to participate/unique first survey page visitors)	Count the unique number of people who filled in the first survey page (or agreed to participate, for example by checking a checkbox), divided by visitors who visit the first page of the survey (or the informed consents page, if present). This can also be called "recruitment" rate.	40/50
	Completion rate (Ratio of users who finished the survey/users who agreed to participate)	The number of people submitting the last questionnaire page, divided by the number of people who agreed to participate (or submitted the first survey page). This is only relevant if there is a separate "informed consent"	24/40

	Checklist for Reporting Results of Internet E-Surveys (CHERRIES)		
Item Category	***Checklist Item***	***Explanation***	***Author Response***
		page or if the survey goes over several pages. This is a measure for attrition. Note that “completion” can involve leaving questionnaire items blank. This is not a measure for how completely questionnaires were filled in. (If you need a measure for this, use the word “completeness rate”.)	
Preventing multiple entries from the same individual			
	Cookies used	Indicate whether cookies were used to assign a unique user identifier to each client computer. If so, mention the page on which the cookie was set and read, and how long the cookie was valid. Were duplicate entries avoided by preventing users access to the survey twice; or were duplicate database entries having the same user ID eliminated before analysis? In the latter case, which entries were kept for analysis (eg, the first entry or the most recent)?	Use of cookies when entering the page. Access to the survey twice were avoided.

	**Checklist for Reporting Results of Internet E-Surveys (CHERRIES)**		
Item Category	***Checklist Item***	***Explanation***	***Author Response***
	IP check	Indicate whether the IP address of the client computer was used to identify potential duplicate entries from the same user. If so, mention the period of time for which no two entries from the same IP address were allowed (eg, 24 hours). Were duplicate entries avoided by preventing users with the same IP address access to the survey twice; or were duplicate database entries having the same IP address within a given period of time eliminated before analysis? If the latter, which entries were kept for analysis (eg, the first entry or the most recent)?	n/a
	Log file analysis	Indicate whether other techniques to analyze the log file for identification of multiple entries were used. If so, please describe.	n/a
	Registration	In “closed” (non-open) surveys, users need to login first and it is easier to prevent duplicate entries from the same user. Describe how this was done. For example, was the survey never displayed a second time once the user had filled it in, or	No registration, targeted population

	Checklist for Reporting Results of Internet E-Surveys (CHERRIES)		
Item Category	***Checklist Item***	***Explanation***	***Author Response***
		was the username stored together with the survey results and later eliminated? If the latter, which entries were kept for analysis (eg, the first entry or the most recent)?	
Analysis			
	Handling of incomplete questionnaires	Were only completed questionnaires analyzed? Were questionnaires which terminated early (where, for example, users did not go through all questionnaire pages) also analyzed?	Only completed questionnaires were analyzed
	Questionnaires submitted with an atypical timestamp	Some investigators may measure the time people needed to fill in a questionnaire and exclude questionnaires that were submitted too soon. Specify the timeframe that was used as a cut-off point, and describe how this point was determined.	Not measured
	Statistical correction	Indicate whether any methods such as weighting of items or propensity scores have been used to adjust for the non-representative sample; if so, please describe the methods.	n/a
