# Supplementary material for: Implementing Active Assisted Living Technology in the Long-term Care of People Living With Dementia to Address Loneliness: European Survey
Source: JMIR Aging. 2023 Jun 14;6:e45231. doi: 10.2196/45231 (PMC10334712; doi:10.2196/45231)
Supplement: Multimedia Appendix 3 [file aging_v6i1e45231_app3.docx]

| **AAL TECHNOLOGY PER COUNTRY** | | | | | | | | | | | | | | |
| --- | --- | --- | --- | --- | --- | --- | --- | --- | --- | --- | --- | --- | --- | --- |
| **Luxembourg** | **Switzerland** | **Netherlands** | **Norway** | **Germany** | **Belgium** | **Finland** | **France** | **Malta** | **Czechia** | **Slovenia** | **Portugal** | **Greece** | **Bulgaria** | **Serbia** |
| PARO | PARO | PARO | PARO | PARO | PARO | PEPPER | PARO | PARO | PAPERO | PARO | PARO | NINTENDO WII |  | DIGITAL LIFESTORY BOOK |
| JOYFORALL CAT | AIBO | PEPPER | JOYFORALL CAT | JOYFORALL CAT | JOYFORALL CAT | CUDDLER | JOYFORALL CAT | NINTENDO WII | DIGITAL LIFESTORY BOOK | PEPPER | DIGITAL LIFESTORY BOOK | XBOX |  |  |
| DIGITAL LIFESTORY BOOK | PEPPER | NINTENDO WII | AIBO | JOYFORALL DOG | JOYFORALL DOG | DIGITAL LIFESTORY BOOK | DIGITAL LIFESTORY BOOK | XBOX | NINTENDO WII | NINTENDO WII | NINTENDO WII | PLAYSTATION |  |  |
| NINTENDO WII | XBOX | XBOX | JUSTOCAT | JUSTOCAT | JUSTOCAT | XBOX | NINTENDO WII | PLAYSTATION | XBOX | XBOX | XBOX |  |  |  |
| XBOX |  | PLAYSTATION | PEPPER | PEPPER | PEPPER | GUIDE | SMARTMACADAM |  | PLAYSTATION |  | PLAYSTATION |  |  |  |
| PLAYSTATION |  |  | DIGITAL LIFESTORY BOOK | DIGITAL LIFESTORY BOOK | DIGITAL LIFESTORY BOOK |  |  |  |  |  | COGWEB |  |  |  |
|  |  |  | NINTENDO WII | NINTENDO WII | NINTENDO WII |  |  |  |  |  |  |  |  |  |
|  |  |  | XBOX | XBOX | XBOX |  |  |  |  |  |  |  |  |  |
|  |  |  | PLAYSTATION | PLAYSTATION | PLAYSTATION |  |  |  |  |  |  |  |  |  |
|  |  |  | GIRAFF | GIRAFF | TOVERTAFEL |  |  |  |  |  |  |  |  |  |
|  |  |  | KOMP | TOVERTAFEL |  |  |  |  |  |  |  |  |  |  |
|  |  |  | MUSIC DOLL |  |  |  |  |  |  |  |  |  |  |  |
|  |  |  | EASY MUSIC PLAYER |  |  |  |  |  |  |  |  |  |  |  |
|  |  |  | MOTITECH |  |  |  |  |  |  |  |  |  |  |  |

## Multimedia Appendix 3: AAL technology per country
